# Supplementary material for: HPLC-Based Analysis of Impurities in Sapropterin Branded and Generic Tablets
Source: Pharmaceutics. 2020 Apr 3;12(4):323. doi: 10.3390/pharmaceutics12040323 (PMC7237990; doi:10.3390/pharmaceutics12040323)
Supplement: Supplementary file 1 [file pharmaceutics-12-00323-s001.pdf]

pharmaceutics

MDPI

Supplementary Materials: HPLC-Based Analysis of Impurities in Sapropterin Branded and Generic Tablets

Emanuela Scudellaro, Luciana Tartaglione, Fabio Varriale, Carmela Dell'Aversano and Orazio Taglialatela-Scafati[1]

Table S1. Exact masses, molecular formula (MF), Ring Double Bond Equivalents (RDB) and errors (Δppm) measured for $[M+H]^+$ and relevant $[M+Na]^+$ions of each compound contained in Full scan HRMS spectra

Compound	$[M+H]^+$(*m/z*) MF, RDB, (Δppm)	$[M+Na]^+$(*m/z*) MF, RDB, (Δppm)
**BIOPTERIN (1)**	238.0936	260.0755
	$C_9H_{12}N_5O_3$, 6.5, (0.480)	$C_9H_{11}N_5O_3Na$, 6.5, (0.344)
**SEPIAPTERIN (2)**	238.0936	260.0755
	$C_9H_{12}N_5O_3$, 6.5, (0.564)	$C_9H_{11}N_5O_3Na$, 6.5, (0.267)
**7,8-DIHYDROBIOPTERIN (3)**	240.1093	262.0909
	$C_9H_{14}N_5O_3$, 5.5, (0.725)	$C_9H_{13}N_5O_3Na$, 5.5, (-0.154)
**PTERIN (5)**	164.0567	186.0387
	$C_6H_6N_5O$, 6.5, (0.571)	$C_6H_5N_5ONa$, 6.5 (0.478)
**6*R*-TETRAHYDROBIOLUMAZINE (6)**	243.1088	265.0910
**6*S*-TETRAHYDROBIOLUMAZINE (7)**	$C_9H_{15}N_4O_4$, 4.5, (1.022)	$C_9H_{14}N_4O_4Na$, 4.5, (0.995)
**6*S*-TETRAHYDROBIOPTERIN (8)**	242.1248	264.1072
	$C_9H_{16}N_5O_3$, 4.5, (1.752)	$C_9H_{15}N_5O_3Na$, 4.5, (1.815)
**5,6,7,8-TETRABIOPTERIN (9)**	168.0880	190.0700
	$C_6H_{10}N_5O$, 4.5, (0.437)	$C_6H_9N_5ONa$, 4.5, (0.362)

Biopterin (**1**)

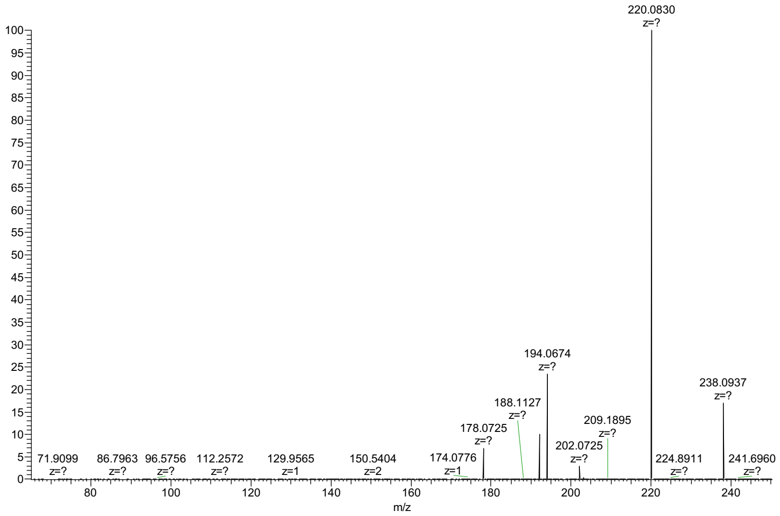

Sepiapterin (**2**)

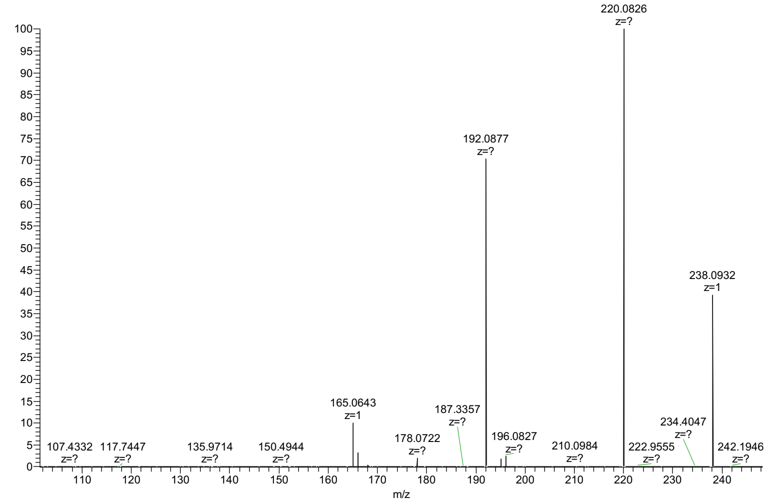

7,8-Dihydrobiopterin (**3**)

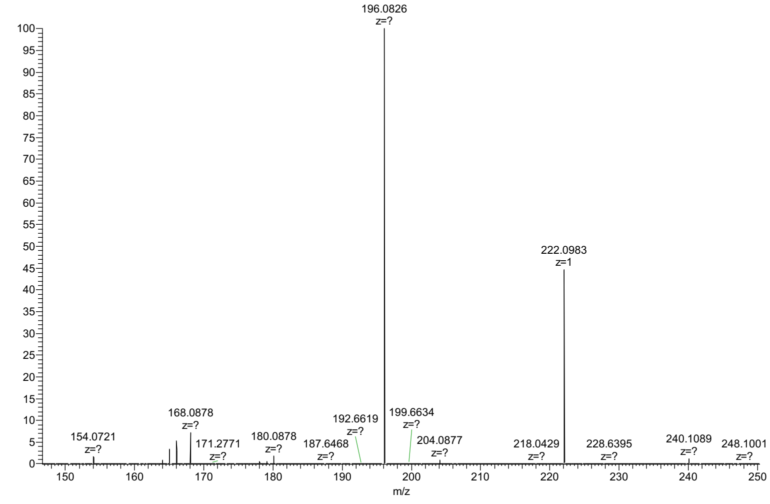

Pterin (**5**)

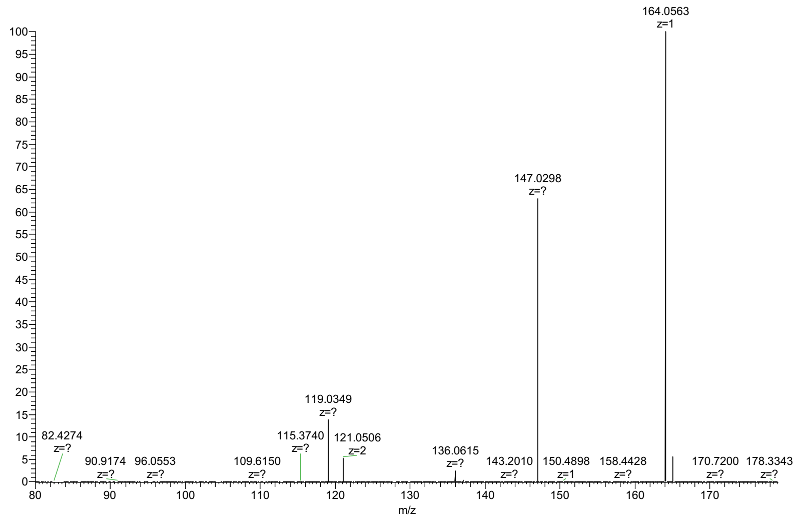

6*R*-tetrahydrobiolumazine (**6**) and 6*S*-tetrahydrobiolumazine (**7**)

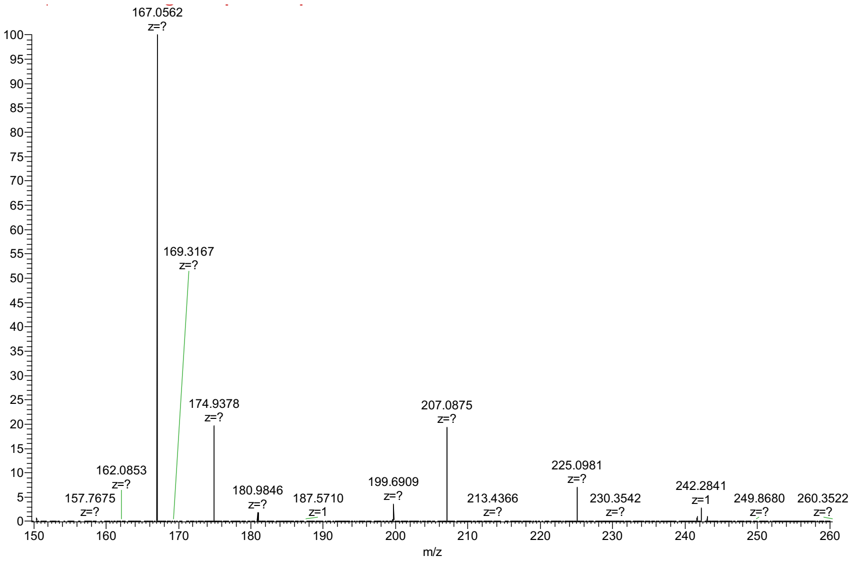

6*S*-tetrahydrobiopterin (**8**)

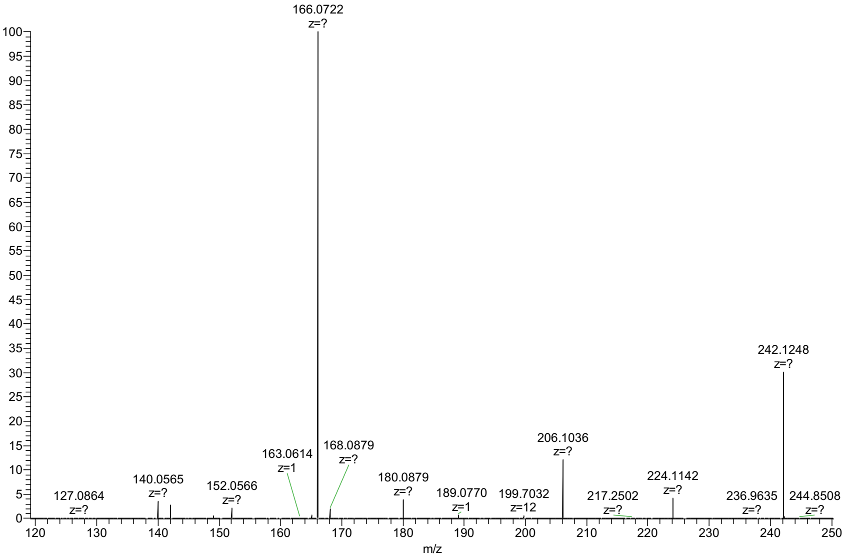

(5,6,7,8)-Tetrahydropterin (**9**)

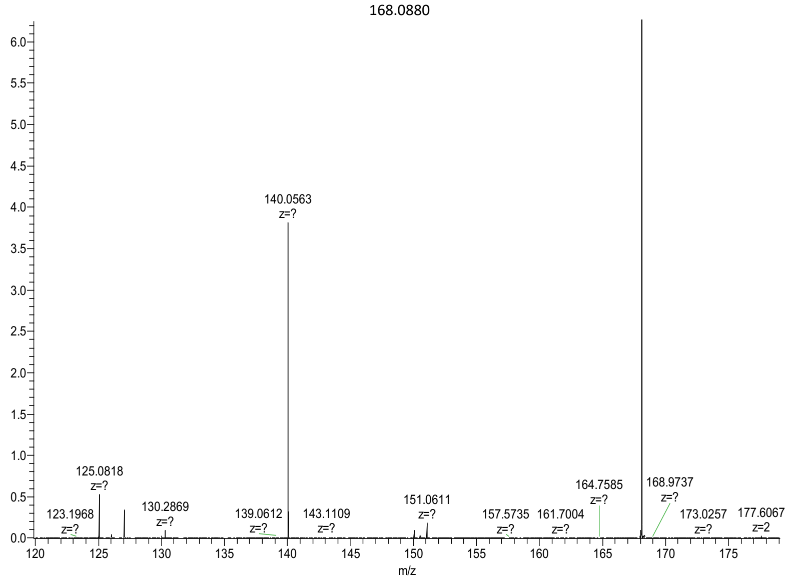

Figure S1. LC-HRMS/MS spectra (CID mode) obtained by selecting the $[M+H]^+$ ion of each impurity/degradation compound as precursor (See Table S1).

Table S2. Exact assignment of the fragment ions contained in the LC-HRMS/MS spectra of compounds **1**-**3** and **5**-**9**. Elemental formulae of the mono-isotopic ion peaks (*m*/*z*) are reported with Ring Double Bond Equivalents (RDB) and errors (Δppm). The most intense fragment ion for each compound is in bold.

***m*/*z*, neutral loss**<br>**MF**<br>**RDB, (Δppm)**													
**Precursor ions**													
**1**		**2**		**3**		**5**		**6/7**		**8**		**9**	
238.0935<br>$C_9H_{12}N_5O_3$<br>6.5, (0.144)		238.0935<br>$C_9H_{12}N_5O_3$<br>6.5, (0.144)		240.1091<br>$C_9H_{14}N_5O_3$<br>5.5, (-0.066)		**164.0567**<br>**$C_6H_6N_5O$**<br>**6.5, (0.083)**		243.1088<br>$C_9H_{15}N_4O_4$<br>4.5, (0.076)		242.1248<br>$C_9H_{16}N_5O_3$<br>4.5, (0.141)		**168.088**<br>**$C_6H_{10}N_5O$**<br>**4.5, (0.080)**	
**Fragment ions**													
**220.083**<br>**$C_9H_{10}N_5O_2$**<br>**7.5, (0.449)**	$-H_2O$	**220.0830**<br>**$C_9H_{10}N_5O_2$**<br>**7.5, (0.449)**	$-H_2O$	222.0986<br>$C_9H_{12}N_5O_2$<br>6.5, (0.220)	$-H_2O$	147.0300<br>$C_6H_3N_4O$<br>7.5, (-0.934)	$-NH_3$	225.0982<br>$C_9H_{13}N_4O_3$<br>5.5, (-0.075)	$-H_2O$	224.114<br>$C_9H_{14}N_5O_2$<br>5.5, (-0.898)	$-H_2O$	151.0612<br>$C_6H_7ON_4$<br>5.5 (-1.572)	$-NH_3$
202.0725<br>$C_9H_8N_5O$<br>8.5, (0.809)	$-2H_2O$	192.0881<br>$C_8H_{10}N_5O$<br>6.5, (0.591)	- HCO_2H	**196.0889**<br>**$C_7H_{10}N_5O_2$**<br>**5.5, (-1.026)**	$-C_2H_4O$	136.0617<br>$C_5H_6N_5$<br>5.5, (-0.528)	-CO	207.0877<br>$C_9H_{11}N_4O_2$<br>6.5, (0.231)	$-2H_2O$	206.1034<br>$C_9H_{12}N_5O$<br>6.5, (-1.148)	$-2H_2O$	140.0565<br>$C_4H_6N_5O$<br>4.5, (-1.331)	$-C_2H_4$
194.0674<br>$C_7H_8N_5O_2$<br>6.5, (0.768)	$-C_2H_4O$	178.0725<br>$C_7H_8N_5O$<br>6.5, (0.919)	- $C_2H_4O_2$	180.0878<br>$C_7H_{10}N_5O$<br>5.5, (-1.036)	- $C_2H_4O_2$	121.0507<br>$C_5H_5N_4$<br>5.5, (-1.427)	-CHNO	**167.0563**<br>**$C_6H_7N_4O_2$**<br>**5.5, (-0.311)**	$-C_3H_8O_2$	189.0769<br>$C_9H_9N_4O$<br>7.5, (-0.992)	$-NH_7O_2$	125.0819<br>$C_5H_9N_4$<br>3.5, (-1.862)	-CHNO
192.0881<br>$C_8H_{10}N_5O$<br>6.5, (0.591)	$-HCO_2H$	165.0643<br>$C_6H_7N_5O$<br>6.0, (-1.281)	- $C_3H_5O_2$	168.0881<br>$C_6H_{10}N_5O$<br>4.5, (0.675)	- $C_3H_4O_2$	119.0349<br>$C_5H_3N_4$<br>6.5, (-2.711)	$-CH_3NO$			180.0878<br>$C_7H_{10}N_5O$<br>5.5, (-1.036)	$-C_2H_6O_2$	127.0612<br>$C_4H_7N_4O$<br>3.5, (-1.868)	$-C_2H_3N$
178.0725<br>$C_7H_8N_5O$<br>6.5, (0.919)	$-C_2H_4O_2$			166.0723<br>$C_6H_8N_5O$<br>5.5, (-0.219)	- $C_3H_6O_2$					168.0878<br>$C_6H_{10}N_5O$<br>4.5, (-1.110)	$-C_3H_6O_2$		
				154.0721<br>$C_5H_8N_5O$<br>4.5, (-1.535)	- $C_4H_6O_2$					**166.0721**<br>**$C_6H_8N_5O$**<br>**5.5, (-1.424)**	$-C_3H_8O_2$		
										165.0644<br>$C_6H_7N_5O$	$-C_3H_9O_2$		

152.0564<br>$C_5H_6N_5O$<br>5.5, (-1.883)	$-C_4H_{10}O_2$
149.0456<br>$C_6H_5N_4O$<br>6.5, (-1.257)	$-C_3H_{11}NO_2$
142.0721<br>$C_4H_8N_5O$<br>3.5, (-1.664)	$-C_5H_8O_2$
140.0564<br>$C_4H_6N_5O$<br>4.5, (-2.045)	$-C_5H_{10}O_2$

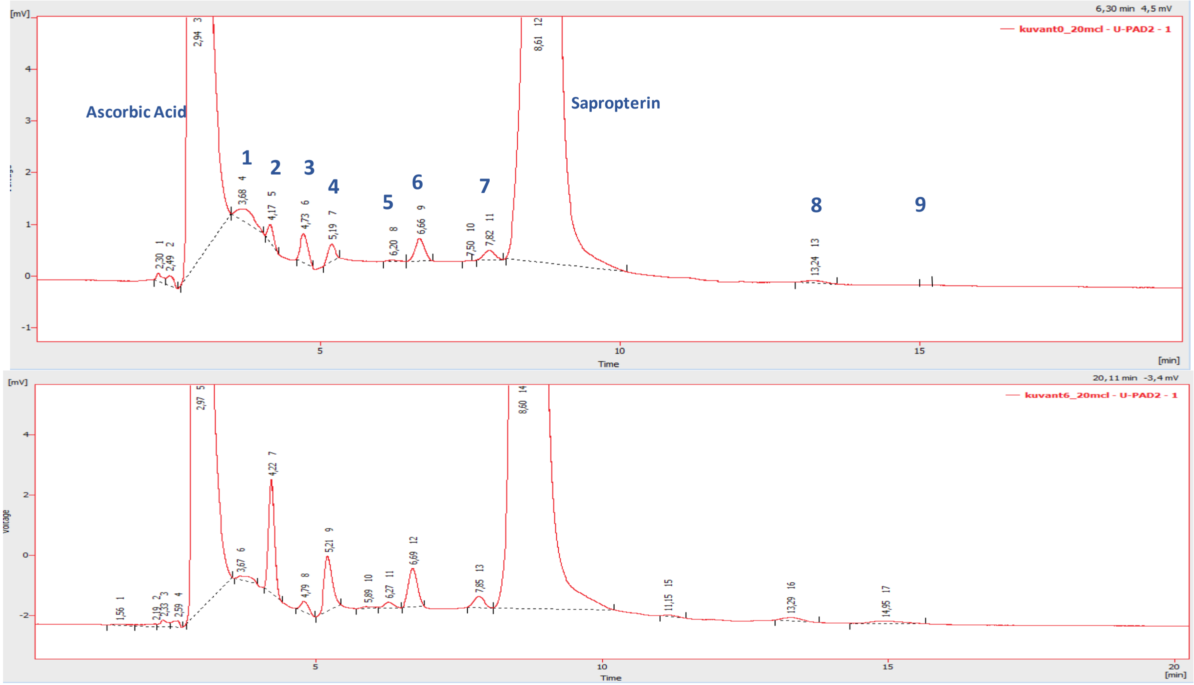

Figure S2. HPLC profile of Kuvan® tablet at T=0 (top) and T = 6 (bottom).

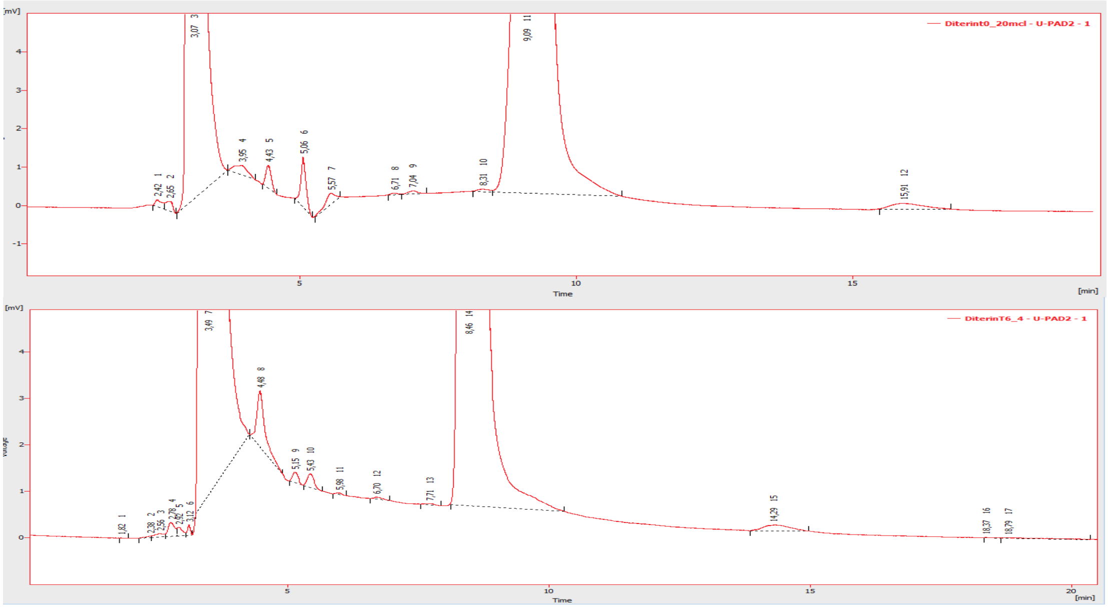

Figure S3. HPLC profile of Diterin tablet at T=0 (top) and T = 6 (bottom).
